# The use of adjuvant chemotherapy for pancreatic cancer varies widely between hospitals: a nationwide population‐based analysis

**DOI:** 10.1002/cam4.921

**Published:** 2016-09-27

**Authors:** Maikel J. Bakens, Lydia G. van der Geest, Magreet van Putten, Hanneke W. van Laarhoven, Geert‐Jan Creemers, Marc G. Besselink, Valery E. Lemmens, Ignace H. de Hingh, the Dutch Pancreatic Cancer Group

**Affiliations:** ^1^ Department of SurgeryCatharina HospitalEindhoventhe Netherlands; ^2^ Netherlands Cancer RegistryNetherlands Comprehensive Cancer Organization (IKNL)Utrechtthe Netherlands; ^3^ Department of Medical OncologyAcademic Medical CenterAmsterdamthe Netherlands; ^4^ Department of OncologyCatharina HospitalEindhoventhe Netherlands; ^5^ Department of SurgeryAcademic Medical CenterAmsterdamthe Netherlands; ^6^ Department of Public HealthErasmus Medical CenterRotterdamthe Netherlands

**Keywords:** Adjuvant chemotherapy, pancreatic cancer, treatment

## Abstract

Adjuvant chemotherapy after pancreatoduodenectomy for pancreatic cancer is currently considered standard of care. In this nationwide study, we investigated which characteristics determine the likelihood of receiving adjuvant chemotherapy and its effect on overall survival. The data were obtained from the Netherlands Cancer Registry. All patients alive 90 days after pancreatoduodenectomy for M_0_‐pancreatic cancer between 2008 and 2013 in the Netherlands were included in this study. The likelihood to receive adjuvant chemotherapy was analyzed by multilevel logistic regression analysis and differences in time‐to‐first‐chemotherapy were tested for significance by Mann–Whitney *U* test. Overall survival was assessed by Kaplan–Meier method and Cox regression analysis. Of the 1195 patients undergoing a pancreatoduodenectomy for pancreatic cancer, 642 (54%) patients received adjuvant chemotherapy. Proportions differed significantly between the 19 pancreatic centers, ranging from 26% to 74% (*P* < 0.001). Median time‐to‐first‐chemotherapy was 6.7 weeks and did not differ between centers. Patients with a higher tumor stage, younger age, and diagnosed more recently were more likely to receive adjuvant treatment. The 5‐year overall survival was significantly prolonged in patients treated with adjuvant chemotherapy—23% versus 17%, log‐rank = 0.01. In Cox regression analysis, treatment with adjuvant chemotherapy significantly prolonged survival compared with treatment without adjuvant chemotherapy. The finding that elderly patients and patients with a low tumor stage are less likely to undergo treatment needs further attention, especially since adjuvant treatment is known to prolong survival in most of these patients.

## Introduction

Pancreatic cancer has a very poor prognosis. Currently, surgical resection is the only possible treatment to obtain long‐term survival 1. The recent CONKO‐001 randomized clinical trial has demonstrated an additional benefit of adjuvant chemotherapy on disease‐free and overall survival for pancreatic cancer 2. These results were obtained in all age groups, for both sexes and independent of tumor stage 2. Given these results, adjuvant chemotherapy is now considered standard of care in most countries including the Netherlands, where adjuvant chemotherapy (Gemcitabine) has been recommended by the Dutch society of Medical Oncology (NVMO) since 2008 3.

In the Netherlands, surgery for pancreatic cancer is only performed in centers performing at least 20 pancreatoduodenectomies (PD) annually. This centralization significantly improved outcomes of pancreatic surgery in terms of postoperative morbidity and mortality 4, 5. In contrast, systemic treatment of pancreatic cancer patients, including adjuvant chemotherapy in operated patients is given in almost all hospitals in the Netherlands. Previous studies have shown that a considerable amount of patients do not receive adjuvant chemotherapy after recovery from a pancreatoduodenectomy 6, 7, 8. It is currently unknown which factors determine the likelihood for receiving adjuvant chemotherapy. Therefore, this nationwide study investigated the variation between pancreatic centers in adjuvant treatment and which characteristics determine the likelihood of receiving adjuvant chemotherapy in the Netherlands. By doing so, correctable reasons for underutilization of adjuvant chemotherapy may be identified, thereby raising the possibility to further improve the treatment of pancreatic cancer patients.

## Patients and Methods

### Data collection

Data were obtained from the nationwide Netherlands Cancer Registry (NCR). This registry contains data of all newly diagnosed cancer patients in the Netherlands (approximately 16.8 million inhabitants in 2013), which is routinely extracted from the medical records in all hospitals and registered by specially trained, independent administrators. The NCR contains patient, tumor, and treatment characteristics. The extent of disease was defined by pathological findings, and was staged using the TNM classification or pathologic extent of disease (pEoD). pEoD classifications were converted to TNM classification 9, 10. In pEoD classification, tumor involvement of the truncus coeliacus or arteria mesenterica superior (AMS) is not specified. Therefore, no differentiation between TNM stage II or III could be made, and these patients were categorized as TNM II/III.

### Patient selection

All nonmetastatic (M_0_) patients diagnosed with adenocarcinoma of the pancreas (ICD C25) 11 between 1 January 2008 and 31 December 2013 in the Netherlands and surgically treated by PD in a pancreatic center were included in this study. Patients diagnosed with carcinoma‐in‐situ (Tis), neuroendocrine tumors, patients with missing data on tumor stage, and patients deceased within 90 days after surgical treatment were excluded from further analysis (*n* = 218). This landmark at 90 days, postoperative, was chosen to minimize the possible effect of postoperative complications on the administration of adjuvant chemotherapy and to deal with immortal time bias of patients receiving chemotherapy. Adjuvant chemotherapy was defined as any chemotherapeutical treatment starting within 16 weeks after surgery.

### Pancreatic center

In the Netherlands, a minimum of 20 PDs per year is currently required to be considered as a pancreatic center. This resulted in 19 pancreatic centers in the Netherlands in 2013, including eight university hospitals.

### Statistical analysis

Differences in patient‐ and tumor characteristics between patients who underwent adjuvant chemotherapy and patients who did not were compared with chi‐square tests. To analyze the hierarchically structured data of patients nested within pancreatic centers, a multilevel logistic regression analysis was used. Multilevel regression analyses provide more accurate estimates when dealing with hierarchically structured data than traditional regression analyses as they account for dependency of patients within pancreatic centers 12, 13. The outcome variable was adjuvant chemotherapy (0, no; 1, yes). Patient‐ and tumor‐related variables (sex, age, TNM stage, year of diagnosis) were added to the multivariable multilevel model. The effect of a variable on the likelihood of adjuvant chemotherapy was expressed as an odds ratio (OR) with 95% Confidence Interval (CI).

Each patient's adjusted chance to undergo adjuvant chemotherapy was given by the following formula: *P* = eL⁄(1 + eL), where L is the calculated value from the logistic regression for that particular patient. The mean adjusted probability to undergo adjuvant chemotherapy for each pancreatic center was defined as the mean adjusted surgical probability of the patients within that pancreatic center. This resulted in a range of probabilities to undergo adjuvant chemotherapy adjusted for differences in patient‐ and tumor characteristics between pancreatic centers. The variation in adjuvant chemotherapy probabilities between pancreatic centers was tested for statistical significance by means of ANOVA with Bonferroni correction.

The differences in comparisons made for the time period between surgery and start of adjuvant chemotherapy, defined as time to adjuvant chemotherapy in weeks, were tested for significance using the nonparametric Mann–Whitney *U* test.

#### Conditional survival

Data retrieved from the Municipal Personal Records Database (BRP) were used to calculate survival. In the BRP, all deaths or emigrations of Dutch inhabitants are registered. Survival time was defined as time from diagnosis to death, or until 1 January 2015 for patients who were still alive. The Kaplan–Meier method was used to determine 5‐year survival. The effect of the time to adjuvant chemotherapy on the overall survival was assessed by log‐rank test. Multivariable Cox regression analysis was undertaken to investigate the prognostic impact of adjuvant chemotherapy on overall survival, after adjustment for patient characteristics. Results from survival analyses using Cox regression analysis were reported as hazard ratios (HR) with 95% CI.

All analyses were performed using Statistical Analysis Software (SAS) version 9.4, North Carolina, USA and a *P* < 0.05 was considered statistically significant.

## Results

### Patients

Between 2008 and 2013, 5846 patients were diagnosed with M_0_‐pancreatic cancer in the Netherlands of whom 1413 (24%) underwent PD in a pancreatic center. In total, 218 patients were excluded. The main reasons for exclusion were diagnosis of a neuroendocrine tumor (*n* = 78) and death within 90 days after surgery (*n* = 84). The remaining 1195 patients were included in this study. Adjuvant chemotherapy was administered to 642 (54%) of these patients, either in the pancreatic center where the surgery was performed (56%) or in the referring hospital (44%). Baseline characteristics differed between patients treated with and without adjuvant chemotherapy, with patients receiving chemotherapy being younger (median 64 vs. 70 years, respectively, *P* < 0.001) and being diagnosed with a higher TNM tumor stage (Table 1).

**Table 1:** Baseline characteristics of M_0_‐pancreatic cancer patients treated by pancreatoduodenectomy between 2008 and 2013 in the Netherlands

Variables	*n* = 1195	Adjuvant chemotherapy	No adjuvant chemotherapy	*P*‐value
		*n* = 642 (54%)	*n* = 553 (46%)	
Sex				
Male	615 (51%)	329 (51%)	286 (52%)	0.871
Female	580 (49%)	313 (49%)	267 (48%)	
Age				
<60 years	285 (24%)	201 (31%)	84 (15%)	<0.001
60–75 years	715 (60%)	409 (64%)	306 (55%)	
≥75 years	195 (16%)	32 (5%)	163 (30%)	
TNM stage				
I	157 (13%)	54 (8%)	103 (19%)	<0.001
II/III	1,038 (87%)	588 (92%)	450 (81%)	
Year of diagnosis				
2008	129 (11%)	42 (7%)	87 (16%)	<0.001
2009	162 (14%)	87 (13%)	75 (13%)	
2010	162 (14%)	84 (13%)	78 (14%)	
2011	188 (15%)	98 (15%)	90 (16%)	
2012	278 (23%)	164 (26%)	114 (21%)	
2013	276 (23%)	167 (26%)	109 (20%)	

### Center of surgery

**Figure 1:**
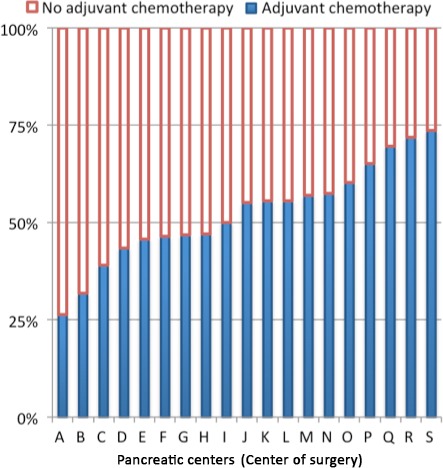
Observed percentage of adjuvant chemotherapy treatment in pancreatic cancer patients undergoing pancreatoduodenectomy in pancreatic centers between 2008 and 2013 in the Netherlands.

**Figure 2:**
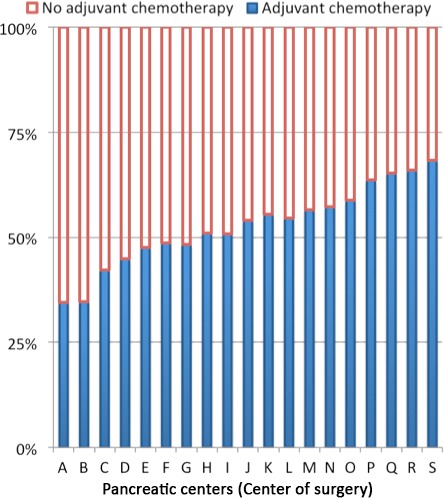
Multilevel case‐mix adjusted probability for adjuvant chemotherapy treatment for pancreatic centers in the Netherlands between 2008 and 2013.

The observed proportion of patients receiving adjuvant chemotherapy differed significantly between the 19 pancreatic centers in the Netherlands and ranged from 26% to 74%, *P* < 0.001 (Fig. 1). Multilevel logistic regression confirmed the effect of the pancreatic center on the probability to undergo adjuvant chemotherapy. The case‐mix adjusted probability for adjuvant chemotherapy treatment ranged between 35% and 68% according to the pancreatic centers (Fig. 2; *P* < 0.001).

No significant difference was found in the observed treatment percentages between university pancreatic centers and nonuniversity pancreatic centers (55% vs. 52%, *P* = 0.245).

Variables influencing the likelihood of receiving adjuvant chemotherapy are presented in Table 2. Multilevel logistic regression model showed that an increased likelihood of adjuvant treatment was observed in patients with a TNM tumor stage II or III compared to TNM stage I (respectively, 57% vs. 34%, OR 2.71, 95% CI: 1.77–4.15). Furthermore, patients older than 60 years were less likely to undergo adjuvant chemotherapy (70% <60 years vs. 57% 60–75 years, OR 0.48, 95% CI: 0.34–0.67). Patients older than 75 years were the least likely to receive chemotherapy (16%, OR 0.06, 95% CI: 0.04–0.10).

**Table 2:** Multilevel logistic regression analyses for the likelihood of adjuvant chemotherapy treatment among M_0_‐pancreatic cancer patients diagnosed between 2008 and 2013 and surgically treated by pancreatoduodenectomy in the Netherlands

Variable	Adjuvant chemotherapy*N* = 642 (54%)	Odds ratio	95% CI
Sex			
Male	329 (54%)	1	
Female	313 (54%)	1.06	0.81–1.40
Age			
<60 years	201 (71%)	1	
60–75 years	409 (57%)	0.48^a^	0.34–0.67
75 years	32 (16%)	0.06^a^	0.04–0.10
TNM stage			
I	54 (34%)	1	
II/III	588 (57%)	2.71^a^	1.77–4.15
Year of diagnosis			
2008	42 (33%)	1	
2009	87 (54%)	2.83^a^	1.61–4.98
2010	84 (52%)	2.85^a^	1.61–5.05
2011	98 (52%)	3.42^a^	1.96–5.99
2012	164 (59%)	4.39^a^	2.59–7.46
2013	167 (61%)	4.63^a^	2.73–7.87

Corrected for pancreatic center, intercept 0.275, SE 0.127.

a Significantly different

Over time, the use of adjuvant chemotherapy increased from 33% in 2008 to 61% in 2013. Patients diagnosed in the year 2013 were more likely to undergo adjuvant treatment compared to patients diagnosed in 2008 (OR 4.63, 95% CI: 2.73–7.87).

### Time to adjuvant chemotherapy

In 400 (62%) patients, adjuvant chemotherapy was initiated within 8 weeks after PD, in 134 (21%) patients, between 8 and 12 weeks postoperatively, and in 23 (4%) patients, treatment was started more than 12 weeks after PD. In 85 (13%) patients, information on time to adjuvant chemotherapy was missing. Median time to adjuvant chemotherapy was 6.6 weeks (Interquartile range [IQR]: 2.9). The time to adjuvant chemotherapy did not significantly differ between patients resected in university centers versus nonuniversity centers, *P* = 0.803 (respectively, median 6.7, IQR: 2.7 vs. median: 6.4, IQR: 3.3). Furthermore, no difference in time to adjuvant chemotherapy was found for patients treated in a pancreatic center versus patients referred to a nonpancreatic center for receiving adjuvant chemotherapy, *P* = 0.194 (respectively, median: 6.3, IQR: 2.9 vs. median: 7.0, IQR: 3.4).

### Conditional survival

**Figure 3:**
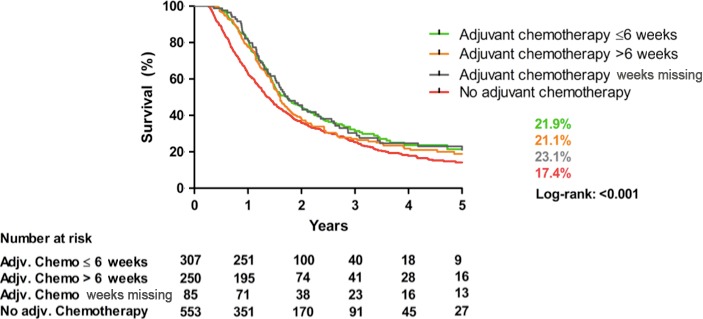
Kaplan–Meier, 5‐year overall survival adjuvant chemotherapy versus no adjuvant chemotherapy after pancreatoduodenectomy in pancreatic cancer patients in the Netherlands between 2008 and 2013.

Kaplan–Meier analysis (Fig. 3) revealed a significant difference in 5‐year overall survival rates based on whether patients were treated by adjuvant chemotherapy—23% versus 17% if not treated by adjuvant chemotherapy (Log‐rank < 0.001). Patients treated with adjuvant chemotherapy had a 5‐year survival rate of 22% if time to adjuvant chemotherapy was ≤6 weeks versus 21% for time to adjuvant chemotherapy >6 weeks. In Cox regression analyses (Table 3), adjuvant chemotherapy treatment was a significant predictor of prolonged survival for both adjuvant chemotherapy within 6 weeks as well as for adjuvant chemotherapy after 6 weeks compared with no adjuvant chemotherapy (HR: 0.68, 95% CI: 0.56–0.82 vs. HR: 0.79, 95% CI: 0.66–0.95). A tumor stage TNM II/III was a significant variable for shortened survival (HR: 1.97 95% CI: 1.58–2.47).

**Table 3:** Cox regression analyses among M_0_‐pancreatic cancer patients diagnosed between 2008 and 2013 in the Netherlands and surgically treated by pancreatoduodenectomy

Variable	Hazard ratio	95% CI
Sex		
Male	1	
Female	0.93	0.82–1.07
Age		
<60 years	1	
60–75 years	1.06	0.90–1.26
≥75 years	1.16	0.92–1.47
TNM stage		
I	1	
II/III	1.97*	1.58–2.47
Year of diagnosis		
2008	1	
2009	0.70*	0.54–0.91
2010	0.90	0.69–1.16
2011	0.87	0.67–1.12
2012	0.93	0.72–1.18
2013	1.10	0.85–1.43
Adjuvant chemotherapy		
No	1	
Yes (started ≤6 weeks postoperative)	0.68*	0.56–0.82
Yes (started >6 weeks postoperative)	0.79*	0.67–0.95
Yes (date of start missing)	0.71*	0.54–0.93

## Discussion

The current population‐based study revealed that 54% of the pancreatic cancer patients received adjuvant chemotherapy following PD. Elderly patients were less likely to undergo adjuvant chemotherapy. Interestingly, the likelihood of receiving adjuvant chemotherapy treatment varied significantly between pancreatic centers. Survival analyses showed that the addition of adjuvant chemotherapy was associated with a prolonged survival. This was seen in patients receiving adjuvant chemotherapy within 6 weeks postoperatively but also in patients receiving chemotherapy more than 6 weeks after PD.

Our findings on overall survival are in line with a recent RCT (randomized clinical trial) and a previous population‐based study in the USA showing a positive influence of adjuvant chemotherapy on overall survival 2, 6. This again stresses the beneficial effect of treating patients with adjuvant chemotherapy, if possible. A recent study in the Netherlands showed limited compliance to quality indicators in pancreatic cancer care based on the Dutch guideline. The administration of adjuvant chemotherapy increased from 45% of patients in 2010 to 54% in 2012 14. Nevertheless, the proportion of patients treated by adjuvant chemotherapy in this study is comparable to percentages described in literature. Mayo et al. 6 reported adjuvant treatment in 51% of patients undergoing any type of surgery for pancreatic adenocarcinoma in Medicare beneficiaries in the USA. A multicenter study in Japan demonstrated that 66% of the pancreatic cancer patients received adjuvant chemotherapy 15. Finally, a study by Aloia et al. 7 showed the highest percentage: 74% of patients received adjuvant therapy after PD. However, in spite of this high percentage, the authors suggested that at least 90% of patients with localized pancreatic adenocarcinoma and good pretreatment performance status would have been candidates for postoperative adjuvant therapy.

A similar limited use of adjuvant chemotherapy has been shown in other tumors. For instance, only 60% patients with colon cancer and lymph node metastases received adjuvant chemotherapy in the Netherlands 16.

Remarkably, the proportion of patients receiving adjuvant chemotherapy varied significantly between pancreatic centers in this study. This finding was not in line with expectations, as all pancreatic centers are supposed to have expert knowledge in the treatment of pancreatic cancer and to adhere to the national guidelines. The differences in the probability to receive adjuvant chemotherapy remained present after adjustment for available case‐mix variables; sex, age, TNM stage, year of diagnosis. There may be various explanations for this phenomenon. First of all, the multidisciplinary tumor boards (MDTB) in the 19 pancreatic centers may have various attitudes toward the guideline recommendations, resulting in a different tendency to advice adjuvant chemotherapy. Since a significant proportion of the patients (44%) were not treated in the pancreatic center but in the referring hospital, medical oncologists from referring hospitals may choose to react differently on the advice of the MDTB. Furthermore, it should be acknowledged that in some cases, patients choose to not undergo adjuvant chemotherapy. This decision‐making process will be the subject of further research.

In this study, age was an important variable in selecting patients with older patients being less likely to receive adjuvant chemotherapy. Previous retrospective studies have reported also an effect of age on the selection of patients for adjuvant chemotherapy 8, 17. However, it was shown in the CONKO‐001 trial that the beneficial effects of adjuvant chemotherapy were obtained regardless of age 2. Also in the cohort study by Nagrial et al., 17 it was demonstrated that adjuvant chemotherapy in elderly patients was associated with an improved survival to at least a similar degree as for younger patients. Furthermore, it is known that PDs can be safely performed in elderly patients with good postoperative outcomes 18, 19. Therefore, physicians may be too reluctant in prescribing adjuvant chemotherapy to elderly patients.

Patients diagnosed with a tumor stage TNM II or III, were more likely to receive adjuvant chemotherapy treatment as compared to patients with stage I disease. Given the worse prognosis in stage TNM II or III patients, especially in the case of lymph node metastases, treating physicians may be more willing to administer adjuvant chemotherapy in these patients. However, as was shown by Oettle and colleagues, the beneficial results of adjuvant chemotherapy were not only achieved in high‐staged tumors but also in low‐staged tumors 2. Therefore, adjuvant chemotherapy treatment of patients with stage I disease needs further attention.

This study had some limitations. Although the NCR registry is a reliable and complete database, data like resection status (R0/R1), postoperative complications, comorbidities, and performance status are lacking. These factors may have influenced the likelihood of receiving adjuvant chemotherapy treatment. Insurance status is not likely to affect the likelihood for adjuvant chemotherapy because of the equally accessible health care system in the Netherlands. Data on type of chemotherapy and completion rates in patients undergoing adjuvant chemotherapy were not registered. In our study, an effort to minimize the possible effect of postoperative complications on the administration of adjuvant chemotherapy was undertaken by excluding patients deceased within 90 days. A correlation between severe complications and omission of adjuvant treatment was reported earlier by Wu et al., 8. Furthermore, they described a decreased likelihood for adjuvant chemotherapy if the length of postoperative stay exceeded 9 days 8. The results of that study showed that withdrawal of adjuvant chemotherapy in some cases could be explained by a prolonged postoperative recovery were early initiation of adjuvant chemotherapy could not be achieved caused by postoperative complications 6, 7, 8. However, recently, Valle et al. 20 reported that survival following start of adjuvant chemotherapy treatment within 8‐12 weeks postoperatively did not differ from initiation within 8 weeks postoperatively. Completion of the full course of the treatment was a more important factor determining outcomes. Likelihood of completion of the full course was maximized by an adequate postoperative recovery. Consequently, an inability of administering adjuvant chemotherapy prior to 8 weeks postoperatively does not eliminate the beneficial effect of chemotherapy, as was confirmed by our study 20. The observed median time of 6.6 weeks between PD and initiation of adjuvant chemotherapy, however, suggests that there might have been a nihilistic approach to a late start of adjuvant chemotherapy. In summary, there is an underuse of adjuvant chemotherapy for pancreatic cancer in the Netherlands. Even in the last year of this study, only 61% of the patients received adjuvant treatment. Elderly patients were less likely to undergo adjuvant chemotherapy, despite the beneficial effect of such treatment also in this age group. Interestingly, the likelihood of receiving adjuvant chemotherapy treatment varied significantly between pancreatic centers, a finding that may not be explained by case‐mix alone. This finding clearly needs further attention and more research, especially since in this study, treatment with adjuvant chemotherapy resulted in a significantly prolonged overall survival. The Dutch Pancreatic Cancer Project (PACAP) including prospective audit, are used for improvements in the use of adjuvant chemotherapy and other relevant factors in survival for pancreatic cancer care in the Netherlands.

## Conflict of Interest

The authors declare no conflict of interest.

## References

[1] Vincent, A. , J. Herman , R. Schulick , R. H. Hruban , and M. Goggins . 2011 Pancreatic cancer. Lancet 378:607–620. 21620466 10.1016/S0140-6736(10)62307-0 PMC3062508

[2] Oettle, H. , P. Neuhaus , A. Hochhaus , J. T. Hartmann , K. Gellert , K. Ridwelski , et al. 2013 Adjuvant chemotherapy with gemcitabine and long‐term outcomes among patients with resected pancreatic cancer: The CONKO‐001 randomized trial. JAMA 310:1473–1481. 24104372 10.1001/jama.2013.279201

[3] Pancreascarcinoom . 2015 Available at: http://www.oncoline.nl/pancreascarcinoom (last accessed: 15 May 2015).

[4] Gooiker, G. A. , V. E. Lemmens , M. G. Besselink , O. R. Busch , B. A. Bonsing , I. Q. Molenaar , et al. 2014 Impact of centralization of pancreatic cancer surgery on resection rates and survival. Br. J. Surg. 101:1000–1005. 24844590 10.1002/bjs.9468

[5] Lemmens, V. E. , K. Bosscha , G. van der Schelling , S. Brenninkmeijer , J. W. Coebergh , and I. H. de Hingh . 2011 Improving outcome for patients with pancreatic cancer through centralization. Br. J. Surg. 98:1455–1462. 21717423 10.1002/bjs.7581

[6] Mayo, S. C. , M. M. Gilson , J. M. Herman , J. L. Cameron , H. Nathan , B. H. Edil , et al. 2012 Management of patients with pancreatic adenocarcinoma: National trends in patient selection, operative management, and use of adjuvant therapy. J. Am. Coll. Surg. 214:33–45. 22055585 10.1016/j.jamcollsurg.2011.09.022 PMC3578342

[7] Aloia, T. A. , J. E. Lee , J. N. Vauthey , E. K. Abdalla , R. A. Wolff , G. R. Varadhachary , et al. 2007 Delayed recovery after pancreaticoduodenectomy: A major factor impairing the delivery of adjuvant therapy? J. Am. Coll. Surg. 204:347–355. 17324767 10.1016/j.jamcollsurg.2006.12.011

[8] Wu, W. , J. He , J. L. Cameron , M. Makary , K. Soares , N. Ahuja , et al. 2014 The impact of postoperative complications on the administration of adjuvant therapy following pancreaticoduodenectomy for adenocarcinoma. Ann. Surg. Oncol. 21:2873–2881. 24770680 10.1245/s10434-014-3722-6 PMC4454347

[9] Sobin, L. H. , and Wittekind C. 2002 TNM Classification of Malignant Tumours. 6th ed Wiley‐Blackwell, New York.

[10] Sobin, L. H. , M. K. Gospodarowicz , and C. Wittekind . 2009 TNM classification of malignant tumours. 7th ed Wiley‐Blackwell, New York.

[11] Fritz, A. G . 2000 International classification of diseases for oncology: ICD‐O. P 240, 3rd edn World Health Organization, Geneva.

[12] Twisk, J . 2006 Applied multilevel analysis. Cambridge Univ. Press, Cambridge.

[13] Austin, P. C. , J. V. Tu , and D. A. Alter . 2003 Comparing hierarchical modeling with traditional logistic regression analysis among patients hospitalized with acute myocardial infarction: Should we be analyzing cardiovascular outcomes data differently? Am. Heart J. 145:27–35. 12514651 10.1067/mhj.2003.23

[14] van Rijssen, L. B. , L. G. van der Geest , T. L. Bollen , M. J. Bruno , A. van der Gaast , L. Veerbeek , et al. 2015 National compliance to an evidence‐based multidisciplinary guideline on pancreatic and periampullary carcinoma. Pancreatology 16:133–137. 26560441 10.1016/j.pan.2015.10.002

[15] Sata, N. , K. Kurashina , H. Nagai , T. Nagakawa , O. Ishikawa , T. Ohta , et al. 2009 The effect of adjuvant and neoadjuvant chemo(radio)therapy on survival in 1,679 resected pancreatic carcinoma cases in Japan: Report of the national survey in the 34th annual meeting of Japanese Society of Pancreatic Surgery. J. Hepatobiliary Pancreat. Surg. 16:485–492. 19333537 10.1007/s00534-009-0077-7

[16] van der Geest, L. G. , J. E. Portielje , M. W. Wouters , N. I. Weijl , B. C. Tanis , R. A. Tollenaar , et al. 2013 All Nine Hospitals in the Leiden Region of the Comprehensive Cancer Centre The N. Complicated postoperative recovery increases omission, delay and discontinuation of adjuvant chemotherapy in patients with Stage III colon cancer. Colorectal Dis. 15:e582–e591. 23679338 10.1111/codi.12288

[17] Nagrial, A. M. , D. K. Chang , N. Q. Nguyen , A. L. Johns , L. A. Chantrill , J. L. Humphris , et al., Australian Pancreatic Cancer Genome I ; M. Pinese , E. K. Colvin , C. J. Scarlett , A. Chou , J. G. Kench , R. L. Sutherland , L. G Horvath , A. V. Biankin , 2014 Adjuvant chemotherapy in elderly patients with pancreatic cancer. Br. J. Cancer 110:313–319. 24263063 10.1038/bjc.2013.722 PMC3899761

[18] Oguro, S. , K. Shimada , Y. Kishi , S. Nara , M. Esaki , and T. Kosuge . 2013 Perioperative and long‐term outcomes after pancreaticoduodenectomy in elderly patients 80 years of age and older. Langenbecks Arch. Surg. 398:531–538. 23462741 10.1007/s00423-013-1072-7

[19] Adham, M. , L. C. Bredt , M. Robert , J. Perinel , C. Lombard‐Bohas , T. Ponchon , et al. 2014 Pancreatic resection in elderly patients: Should it be denied? Langenbecks Arch. Surg. 399:449–459. 24671518 10.1007/s00423-014-1183-9

[20] Valle, J. W. , D. Palmer , R. Jackson , T. Cox , J. P. Neoptolemos , P. Ghaneh , et al. 2014 Optimal duration and timing of adjuvant chemotherapy after definitive surgery for ductal adenocarcinoma of the pancreas: Ongoing lessons from the ESPAC‐3 study. J. Clin. Oncol. 32:504–512. 24419109 10.1200/JCO.2013.50.7657

